# Validation of a hairy roots system to study soybean-soybean aphid interactions

**DOI:** 10.1371/journal.pone.0174914

**Published:** 2017-03-30

**Authors:** Stephanie C. Morriss, Matthew E. Studham, Gregory L. Tylka, Gustavo C. MacIntosh

**Affiliations:** 1 Roy J. Carver Department of Biochemistry, Biophysics and Molecular Biology, Iowa State University, Ames, Iowa, United States of America; 2 Department of Plant Pathology and Microbiology, Iowa State University, Ames, Iowa, United States of America; Montana State University Bozeman, UNITED STATES

## Abstract

The soybean aphid (*Aphis glycines*) is one of the main insect pests of soybean (*Glycine max*) worldwide. Genomics approaches have provided important data on transcriptome changes, both in the insect and in the plant, in response to the plant-aphid interaction. However, the difficulties to transform soybean and to rear soybean aphid on artificial media have hindered our ability to systematically test the function of genes identified by those analyses as mediators of plant resistance to the insect. An efficient approach to produce transgenic soybean material is the production of transformed hairy roots using *Agrobacterium rhizogenes*; however, soybean aphids colonize leaves or stems and thus this approach has not been utilized. Here, we developed a hairy root system that allowed effective aphid feeding. We show that this system supports aphid performance similar to that observed in leaves. The use of hairy roots to study plant resistance is validated by experiments showing that roots generated from cotyledons of resistant lines carrying the *Rag1* or *Rag2* resistance genes are also resistant to aphid feeding, while related susceptible lines are not. Our results demonstrate that hairy roots are a good system to study soybean aphid-soybean interactions, providing a quick and effective method that could be used for functional analysis of the resistance response to this insect.

## Introduction

The soybean aphid, *Aphis glycines* Matsumura (Hemiptera: Aphididae), is one of the main insect pests of soybean (*Glycine max* L.) worldwide, and it has had a significant impact as an invasive pest on soybean production in North America in the last 16 years [1]. Like other hemipterans, soybean aphids are phloem feeders, taking photosynthates directly from phloem sap. In addition, aphids are viral vectors, and their honeydew favors the growth of sooty mildew on leaves causing reductions in photosynthesis [2, 3]. These direct and indirect effects of aphid feeding can result in soybean yield losses of up to 40% if the pest is not managed [1, 3]. So far, management strategies have been based mainly on insecticide applications [3], which increase production costs and also have negative impacts on the environment. Host plant resistance is starting to be incorporated in management strategies. Several sources of resistance have been identified in cultivated and wild soybean lines [2]. From those sources, eight *Rag* (*Resistance to Aphis glycines*) genes have been identified. Most of these genes have been mapped and their type of resistance, antibiosis or antixenosis, has been characterized (summarized in [1]). Commercial soybean varieties carrying *Rag1*, *Rag2* or a combination of both genes were recently made available to producers [4, 5].

However aphid biotypes that can overcome *Rag* resistance have been identified. Aphids corresponding to Biotype 1 are controlled by plants carrying all the *Rag* genes tested so far; Biotype 2 aphids can overcome *Rag1* resistance but are controlled by the resistance provided by *Rag2*; Biotype 3 is controlled by *Rag1* resistance but not by *Rag2* resistance; and Biotype 4 aphids can overcome plants carrying *Rag1* and *Rag2* resistance, including the *Rag1/Rag2* pyramid, but are controlled by *Rag3* resistance [6–8]. The evolution of aphid biotypes that are not controlled by *Rag* resistance genes, even before strong selective pressure has been exerted in the field, has brought questions to the sustainability of host resistance as management strategy [1, 9], and highlight the need to understand the molecular mechanisms underlying aphid resistance in soybean in order to devise novel aphid control strategies.

Transcriptome analyses have provided some insight on these mechanisms. Comparison of changes in gene expression in response to aphid feeding between *Rag1* lines and susceptible plants showed that the resistance response is established quickly, between 6–12 h after initial feeding, and it is likely controlled by salicylate and jasmonate signaling [10, 11]. On the other hand, susceptible plants have a slower response that cannot prevent potential defense suppression mechanisms elicited by the aphid [11]. Metabolite analyses also suggest that resistant (*Rag1*) plants seem to prevent successful aphid colonization through changes in free amino acid profiles that may affect the nutritional quality of the plant for the aphid [12]. Interestingly, transcriptome analysis of aphids fed on susceptible or *Rag1* plants suggested that chemical defenses may be an important component of the resistance response, as aphids feeding on resistant plants differentially increased expression of detoxifying enzymes [13]. Proteome and transcriptome analyses also implicated changes in primary metabolism and defense mechanisms including cell wall strengthening in *Rag2*-mediated resistance [14].

Although advances have been made in the characterization of the soybean defense responses to aphid feeding, research in this area is hindered by the difficult to generate soybean mutants. Functional analysis of defense gene candidates to mediate resistance to pests are commonly tested through loss- and gain-of-function mutants such as those obtained by overexpressing the candidate gene or silencing its expression using RNAi. Due to the lengthy and complicated processes needed to achieve stable transformation in soybean [15], these approaches have yet to be applied consistently to study soybean-soybean aphid interactions, and may be one of the reasons why none of the *Rag* genes have yet been cloned. An effective alternative to stable transformation to functionally characterize candidate genes is the use of virus-induced gene silencing (VIGS). VIGS vectors based on bean pod mottle virus have been optimized for use with soybean [16], and provide a quick method that can be deployed in high throughput strategies. This approach has been successfully applied to study several systems. For example, VIGS was used to find the identity of the soybean *Rpp4* gene that confers resistance to Asian soybean rust [17] and to characterize components of the gene regulatory network mediating this resistance [18]. A similar approach was used to identify components of the soybean resistance response against soybean mosaic virus (*Phakopsora pachyrhizi* H. Sydow & Sydow) [19], and to characterize the role of *RIN4* in RPG1-B-derived resistance to the bacterium *Pseudomonas syringae* in soybean [20].

While VIGS is an effective approach for functional analysis of candidate genes, it involves infecting plants with a viral vector, which could mask or alter the defense responses under study. Thus, alternative approaches that could complement VIGS would be ideal. One complementary approach is the use of hairy roots [21]. Hairy roots are transgenic tissues that result from *Agrobacterium rhizogenes* infections. This bacterium induces the neoplastic growth of plant cells that differentiate to form hairy roots. Morphologically, *A*. *rhizogenes*-induced hairy roots are very similar in structure to wild-type roots. Hairy roots are induced by the incorporation of a bacterial-derived segment of DNA transferred (T-DNA) into the chromosome of the plant cell; the expression of genes encoded within the T-DNA promotes the development and production of roots at the site of infection on most dicotyledonous plants. A key characteristic of hairy roots is their ability to grow quickly in the absence of exogenous plant growth regulators. As a result, hairy roots are widely used as a transgenic tool for the production of metabolites and for the study of gene function in plants. Researchers have utilized this tool to study root development and root–biotic interactions, to overexpress proteins and secondary metabolites, to detoxify environmental pollutants, and to increase drought tolerance [21]. In soybean, hairy root cultures have been used for propagating obligate soybean root parasites and for testing interactions between soybean and the parasite, especially the soybean cyst nematode (*Heterodera glycines* Ichinohe) [22, 23]. In these cases, since only root tissue is used, plant regeneration is not necessary, and the generation of transgenic hairy roots takes only 4 weeks. However, since the soybean aphid is a leaf/stem herbivore, this approach has not been used to study soybean aphid resistance, and until now it was not known whether soybean aphids could feed and reproduce on hairy roots.

Here, we show that soybean aphids do indeed feed on hairy roots and the aphid population grows similarly as aphids growing on leaves. We also validated this method to study soybean aphid resistance genes. We found that both *Rag1* and *Rag2* resistance are expressed in roots, and colonies of biotype 1 aphids cannot grow on roots of these genotypes. Since these traits are expressed in roots and several lines of research point to crosstalk between aphid and nematode resistance mechanisms, we tested whether these genes also protected soybean plants from soybean cyst nematode infestation. Our results demonstrate that hairy roots is a good system to study soybean-soybean aphid interactions.

## Material and methods

### Plant material and aphid colony

The soybean lines used were LD-16060, an aphid resistant line carrying the *Rag1* gene [12], and its parent line SD01-76R (aphid susceptible), obtained from Dr. B. Diers (University of Illinois, Urbana-Champaign, IL). We also used four related lines developed by Dr. W. Fehr (Iowa State University), one susceptible to aphid infestation and the others carrying the resistance genes *Rag1*, *Rag2*, or a pyramid of both genes (described in [24]), that will be referred as Susc2, Rag1, Rag2, and Rag1/Rag2 respectively throughout this work.

A colony of biotype 1 soybean aphids maintained on the SD01-76R cultivar for several years was used for all the experiments. This colony is kept in a growth chamber at constant temperature (21°C) and with a light regime of 16 h light/ 8 h dark. To perpetuate the colony, every 30 days new plants were infested at the V3 stage by attaching an infested leaf from a colony plant to the new plant, and old plants were eliminated. One week before each experiment, an infested leaf from a colony plant was transferred to a fresh, aphid-free plant kept on a separate chamber under the same environmental conditions as the colony, and aphids from the new plant were used for the experiments.

### Hairy root induction

Hairy root production followed the method by Subramanian et al. [25], with some modifications. Briefly, seeds for hairy root induction were germinated on coarse vermiculite in growth chambers maintained at 21°C under 16 h light/ 8 h dark conditions. One week after sowing, cotyledons were excised and sterilized using wipes soaked with a 70% (v/v) ethanol solution. Cotyledons were then placed onto petri plates (10 per plate) cover with filter paper wetted with 2–3 ml of distilled water. Using a sterile scalpel, four incisions were made on the abaxial surface of the cotyledons. Ten μl of an *Agrobacterium rhizogenes* K599 (pRi2659) culture grown in LB at 28°C for 2 days (approximate O.D. was 1.0–1.5) were applied to the incisions of each cotyledon. Individual cotyledons were placed in petri dishes, on sterile filter paper dampened with deionized water, and maintained at 22–25°C under 18h light/ 6h dark conditions. During growth, hairy root systems remained sealed with parafilm, and water was added as needed to maintain moisture.

### Soybean aphid infestation

#### Hairy roots

Hairy roots were infested once the root network had reached a size capable of sustaining an aphid population (larger root length > 1 inch). Ten adult aptera aphids (7–8 day old) were applied to the larger root below the base of the root. Plates were kept in growth chambers maintained at 25°C under 18h light/ 6h dark conditions. After five or seven days (as indicated in each figure), aphids were counted and the number of aphids on roots or cotyledon recorded. To compare aphid performance between susceptible and resistant lines on roots and leaves, aphid count was normalized to the average number of aphids on susceptible roots or leaves. Again, water was added to the plates as needed to maintain moisture. Two or three independent experiments (including three to ten replicates for each treatment, completely randomized design), as indicated in each figure legend, were analyzed using Student’s *t*-test assuming unequal variance to determine mean differences in pairwise comparisons. p<0.05 was considered statistically significant.

#### Full plants

Plants were grown in growth chambers maintained at 25°C under 18h light/ 6h dark conditions. For full plant infestation, soybeans plants at the V3 growth stage were infested with ten soybean aphid aptera (7–8 day old) on the V3 trifoliate. Plants were covered with nets secured with rubber bands around the pot to keep aphids from moving from plant to plants. After seven days, aphids were counted and normalized to the average aphid number of the susceptible plant. Two independent experiments (including ten replicates for each treatment, completely randomized design), were analyzed using Student’s *t*-test assuming unequal variance, to determine mean differences in pairwise comparisons. p<0.05 was considered statistically significant.

### Soybean Cyst Nematode (SCN) infestation

SCN reproduction was quantified following the method of Niblack et al. [26] for greenhouse evaluation of soybean resistance to SCN. Briefly, soybean seeds of each genotype (SD01-76R, LD-16060, Rag2, and Susc2), Williams 82 (SCN-susceptible control), and Jack (SCN-resistant control) were planted into individual replicate cone-tainers filled with soil-sand mix infested with SCN HG Type 2.5.7 [27] originally obtained from Muscatine, Iowa. Cone-tainers were placed in buckets of sand in a greenhouse water bath and incubated at 27°C under natural and supplemented lighting conditions. After 30 days of incubation, roots from each individual cone-tainer were removed and washed. The roots were subsequently placed on a sieve with 850 μm pores nested over a sieve with 250 μm pores and sprayed with a strong stream of water. The stream of water dislodged the SCN females and cysts from the roots. The SCN females and cysts passed through the top, 850-μm-pore sieve and were collected on the bottom, 250-μm-pore sieve. The SCN females and cysts collected on the bottom sieve and all other debris on that sieve were observed with a dissecting microscope, and the number of SCN females and cysts recovered from each individual plant were counted. Two independent experiments (including ten replicates for each treatment, completely randomized design), were analyzed using one way ANOVA with post-hoc Tukey HSD (p<0.05) for means comparisons.

## Results and discussion

### Soybean aphids feed and reproduce on hairy roots

**Fig 1:**
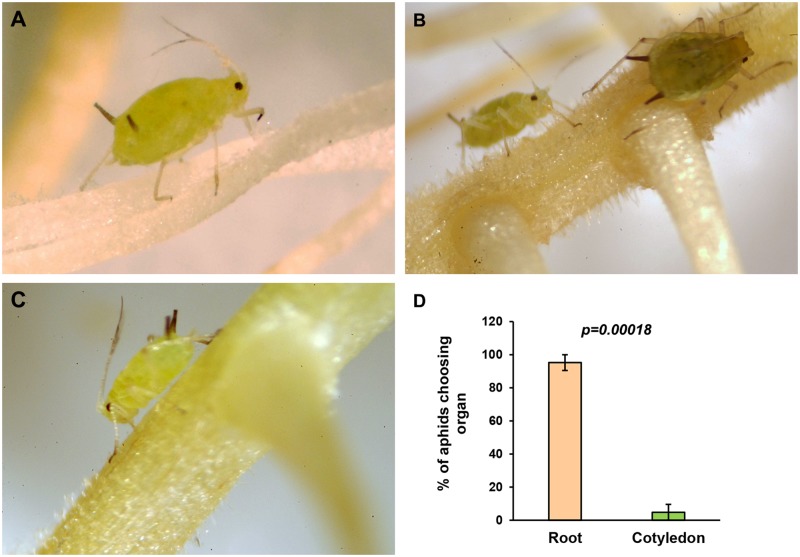
Soybean aphids feed on soybean hairy roots. Aphids were introduced to plates with individual hairy root systems produced by *Agrobacterium rhizogenes* infection of soybean cotyledons. Roots were kept attached to the cotyledon and the whole system was kept on paper filter wetted with water. Aphids fed on young (**A**) or older (**B, C**) roots. **D**. Quantification of aphid feeding preference. After five days of colonization, the number of aphids feeding on roots or cotyledons was recorded, and the percentage of aphids on each organ is reported. Error bars = standard error. Three independent experiments with three replicates each were analyzed using Student’s *t*-test.

Different protocols for hairy root transformation exist, varying in the way cotyledons are infected with *A*. *rhizogenes*, media used, and root maintenance [25, 28–30]. We settled on a method [25] that minimizes contamination by maintaining roots on filter paper soaked in water throughout the length of the experiment. This approach kept roots drier than when they are grown directly on agar, and allowed aphids to walk from root to root freely. Immediately after introduction into hairy root plates, aphid started feeding directly on roots (Fig 1A–1C). There was no obvious preference for any part of the root, and aphids fed on younger (Fig 1A) and older roots (Fig 1B and 1C), which were selected at apparent similar rates. However, aphids clearly avoided the cotyledon, which was not removed from the root system for our experiments. More than 90% of aphids had settle on roots rather than cotyledon after five days (Fig 1D) or seven days (not shown) of colonization. This strong and statistically significant (p<0.0005) preference for root tissues instead of cotyledons is important because while the roots can be transformed with *A*. *rhizogenes* T-DNA, the cotyledon is not; thus, aphid preference for roots eliminates the need for cotyledon removal when the hairy root system is used to produce transgenic roots with introduced or silenced genes.

**Fig 2:**
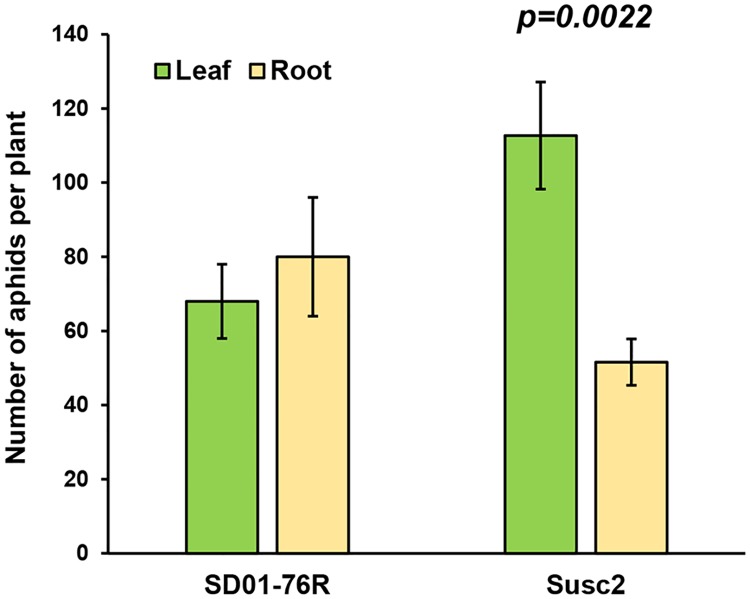
Aphid performance on hairy roots and full soybean plants. Ten aphids were introduced to each plate with one cotyledon and hairy root system, or to the V3 leaf of a soybean plant. After one week, aphid numbers were recorded. Two different aphid-susceptible soybean cultivars were tested, SD01-76R and Susc2. Two independent experiments with 10 replicates for each cultivar and treatment were carried out. Comparison of aphid performance on roots and leaves of each cultivar was performed using Student’s *t*-test. Error bars = standard error.

We then compared soybean aphid performance on hairy roots and on leaves of full plants. We used aphid-susceptible cultivars, infested leaves of pot-grown plants with 10 aphids, and quantified aphid populations on each plant after seven days. Similarly, 10 aphids were introduced on plates with one cotyledon and hairy root system and aphids counted after a similar period of time. We observed that aphid population growth with similar on leaves and hairy roots using the SD01-76R cultivar (Fig 2). A statistically significant difference (p<0.005), with a population about 50% lower on roots than in leaves was observed for the Susc2 line (Fig 2). Compared with the results for SD01-76R, this difference seems to derive from a higher number of aphids in leaves rather than poor performance on hairy roots derived from Susc2. Susc2 was produced from crosses that use the line IA3027 as one of the recurrent parents [24], and it has been reported that IA3027 is highly susceptible to aphid colonization, with populations almost twice the size of those on SD01-76R in some experiments [31]. Thus, it is clear that aphid performance on roots may depend on the genetic background of the soybean cultivar used for hairy root production, even when using susceptible lines.

### Resistance to soybean aphids is expressed in hairy roots

**Fig 3:**
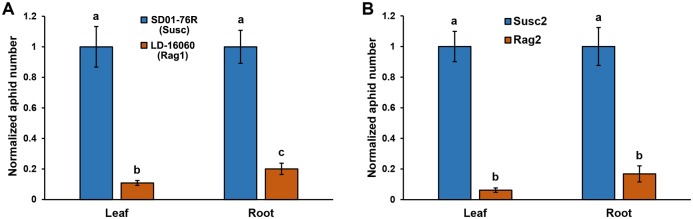
Soybean hairy roots can be used to screen for resistance. **A**. Aphid performance on roots and full plants of a susceptible cultivar (SD01-76R) and a related resistant cultivar (LD-16060) carrying the *Rag1* resistance gene. Full plants and hairy roots were infested with 10 aphids, and the number of aphids after 7 days was recorded. **B**. Aphid performance on roots and full plants of a susceptible cultivar (Susc2) and a related resistant cultivar (Rag2) carrying the *Rag2* resistance gene. Full plants and hairy roots were infested with 10 aphids, and the number of aphids after 7 days was recorded. Two independent experiments with 10 replicates for each cultivar and treatment were carried out. In **A** and **B**, number of aphids for each replicate was normalized to the average number of aphids on the susceptible cultivar for each organ, to facilitate comparison. Statistically significant differences are indicated by different letters above the bars (Student’s *t*-test; p<0.05). Error bars = standard error.

Differences in aphid performance on leaves and on roots of the Susc2 line could possibly indicate that traits important for aphid reproduction or feeding, such as the putative increase in susceptibility observed in IA3027, are not expressed in roots. This would be a significant flaw for a system aimed to dissect the molecular mechanisms of soybean resistance to aphids. To test whether aphid resistance is expressed in roots, we generated hairy roots from LD-16060, which carries the *Rag1* resistance gene, and the related susceptible line SD01-76R, and allowed aphids to colonize roots for seven days. In parallel, we tested aphid performance on both lines using pot-grown plants. Statistically significant differences between susceptible and resistant lines were observed for leaves and roots. We found that *Rag1* resistance was clearly expressed in roots (Fig 3A); and difference in aphid numbers between roots of the susceptible and resistant line were similar to the number difference observed in leaves, although a small but significant difference between leaves and roots of the resistant line were observed (Fig 3A). A similar experiment was performed with the susceptible line Susc2 and the related line Rag2, and almost identical results were obtained (Fig 3B), with statistically significant differences (p<0.05) between susceptible and resistant lines. *Rag2*-dependent resistance was also strongly expressed in roots and in this case the difference in aphid performance between susceptible and resistant line was similar in leaves and roots.

The *Rag1* gene was originally identified in the cultivar Dowling as a single dominant gene trait [32], and it was determined that it confers strong antibiosis-type resistance [32, 33]. Fine mapping has identified a small region of the soybean genome where this gene is located, and two nucleotide binding leucine-rich repeat (NBS-LRR) resistance genes in this region have been suggested as *Rag1* candidates [34]. Similarly, *Rag2* is also a single dominant gene, and it provides strong antibiosis against soybean aphids [35, 36]. Fine mapping of *Rag2* also identified an NBS-LRR candidate as the putative resistance gene [37]. Our root results are consistent with an antibiosis trait. Moreover, our results show that the molecular components of the resistance trait that limit aphid performance, are expressed in the root system. Thus, the hairy root method is an ideal system to dissect the molecular basis of resistance provided by the *Rag1* and *Rag2* genes and potentially other molecular events mediating soybean aphid-soybean interactions. It will be important to extend the characterization of this system to other *Rag* genes to determine its applicability; and to include genes providing antixenosis-type resistance, such as *Rag5* [1, 38], to determine whether hairy roots can also be used in choice experiments that test feeding deterrence.

### *Rag1* and *Rag2* do not provide protection against the soybean cyst nematode

**Fig 4:**
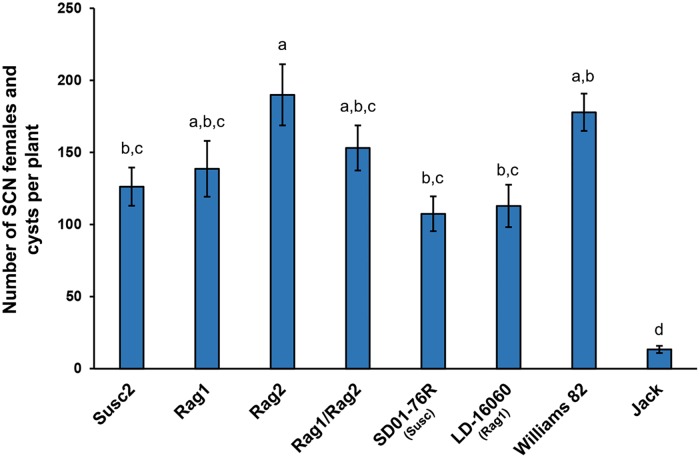
*Rag1* and *Rag2* do not provide protection against the Soybean Cyst Nematode (SCN). Aphid-susceptible (SD01-76R, Susc2) and resistant (LD-16060, Rag1, Rag2, Rag1/Rag2) soybean lines, and SCN susceptible (Williams-82) and resistant (Jack) controls were sowed in soil infested with SCN. Plants were collected after 30 days, and SCN females and cysts were removed from roots and counted. Two independent experiments with 10 plants per line were carried out. Statistically significant differences are indicated by different letters above the bars (One way ANOVA with Tukey HSD; p<0.05). Error bars = standard error.

Few aphid resistance genes have been cloned [39]. Among those, the best characterized is *Mi-1*.*2*, a tomato gene that confers resistance to the potato aphid [39, 40]. This gene also provides resistance against root-knot nematodes, *Meloidogyne* spp. [40], suggesting a crosstalk between nematode and aphid resistance pathways in this plant. In soybean, evidence for crosstalk between aphids and nematode defenses also exist. Soybean cyst nematodes show an increase in reproductive rates when soybean plants are simultaneously colonized by aphid, compared to the rates observed on plants free of aphids [41, 42]. Given that our previous results showed that *Rag1* and *Rag2* resistance is expressed in roots, it could be possible that soybean aphid resistance genes also contribute to the defense against SCN. To test this possibility, we analyzed SCN reproduction on the same aphid susceptible and aphid resistant lines used in our previous experiments, and we included also a line in which *Rag1* and *Rag2* were combined. This Rag1/Rag2 line has been shown to have enhanced resistance to soybean aphid with respect to lines with the individual genes [43]. Against our expectation, nematode numbers were not significantly (p>0.05) lower for resistant lines compared to their related susceptible lines, indicating that none of the aphid resistant lines provided protection against SCN (Fig 4).

## Conclusions

In this work, we were able to show that soybean aphids can feed on and colonize hairy roots generated by *A*. *rhizogenes* transformation. With careful handling we were able to mostly avoid contamination of the root systems. This was in part due to the selection of a method that did not rely on media with sucrose to maintain the roots, since the cotyledon was kept attached to the root system. However, the recent development of “in vitro aphids”, aphids reared in sterile conditions and free of contaminating microorganisms [44], opens the possibility of using media with sugar or other additions if researchers deem it necessary for their experiments.

We also demonstrated that *Rag1*- and *Rag2*-derived resistance are expressed in hairy roots, opening the possibility to utilize this system to study the molecular mechanisms that provide resistance against the insect. These resistance genes have been mapped to small regions of the soybean genome, which include promising candidate genes [34, 37], yet confirmation on the participation of these candidates in resistance is still missing. Hairy roots could be a good approach to incorporate these NBS-LRR candidate resistance genes that map to the *Rag1* and *Rag2* loci into a susceptible cultivar to confirm their involvement in resistance. This system could also be used to dissect the involvement of classical signaling components, such as proteins participating in pattern-triggered immunity, effector-triggered immunity, and phytohormone signaling modules (reviewed in [39, 45]), in the resistance pathways.
